# Comparison of C-Reactive Protein and Serum Amyloid A Protein in Septic Shock Patients

**DOI:** 10.1155/2008/631414

**Published:** 2008-03-12

**Authors:** Domingos Dias Cicarelli, Joaquim Edson Vieira, Fábio Ely Martins Benseñor

**Affiliations:** Divisão de Anestesia, Departamento de Cirurgia, Hospital das Clínicas da Faculdade de Medicina da Universidade de São Paulo, 01246-903 São Paulo, SP, Brazil

## Abstract

Septic shock is a severe inflammatory state caused by an infectious agent. Our purpose was to investigate serum amyloid A (SAA) protein and C-reactive protein (CRP) as inflammatory markers of septic shock patients. Here we evaluate 29 patients in postoperative period, with septic shock, in a prospective study developed in a surgical intensive care unit. All eligible patients were monitored over a 7-day period by sequential organ failure assessment (SOFA) score, daily CRP, SAA, and lactate measurements. CRP and SAA strongly correlated up to the fifth day of observation but were not good predictors of mortality in septic shock.

## 1. INTRODUCTION

Severe sepsis and septic shocks are a common cause of mortality in intensive care unit (ICU) [1]. They are a state of systemic inflammation in response to infectious agents that can lead to multiple organ system failure and death.

The systemic inflammatory response to infection involves the release of several mediators, which has led to the suggestion that some of these mediators could be used as markers of sepsis severity [2]. Among the acute-phase proteins that participate in the inflammatory response, C-reactive protein (CRP) is a component of the innate immune system that binds phosphocoline and recognizes some foreign pathogens as well as phospholipid constituents of damage cells; serum amyloid A (SAA) protein is an apolipoprotein that rapidly binds to high-density lipoprotein after their synthesis, influencing cholesterol metabolism during inflammatory states, causing adhesion and chemotaxis of phagocitic cells and lymphocytes [3, 4]. In some patients with chronic inflammation, the net effect of increased SAA production may be deleterious due to tissue deposition of its fragments and the development of systemic amyloidosis [3, 5].

CRP and SAA display a similar pattern in most inflammatory diseases, reaching a maximum serum concentration about 24 hours after the inflammatory process sets in and slowly decreasing [6]. CRP is commonly used as a marker of an acute inflammatory state, produced by the liver in response to tissue injury or infection [7]. Its plasma concentration has been reported to parallel the clinical course of infection and the fall of the protein level indicates the resolution of infection [1]. SAA is the other major acute-phase protein in humans, with the earliest and highest increase rate of all acute-phase proteins, including CRP [4, 8]. SAA concentrations usually parallel those of CRP. Some authors have been reported that SAA appears to be a clinically useful marker of inflammation in bacterial or viral infection likewise CRP [9]. Although some studies suggest that SAA is a more sensitive marker of inflammatory disease, assays for SAA are not widely available at present [4].

Until now, no study has compared daily CRP to SAA plasma concentrations in postoperative patients with septic shock, or has correlated them to the severity of patients represented by SOFA score. This study aimed to evaluate CRP and SAA measurements as markers of severity of septic shock patients during postoperative period.

## 2. METHODS

This study was prospective at a surgical ICU. After approval by a local ethics committee, informed consent was obtained from patients or from their next of kin prior to enrollment [10]. Twenty-nine patients admitted into the surgical ICU of the Hospital das Clínicas da Faculdade de Medicina da Universidade de São Paulo had taken part in the study. Additional three patients were excluded after their next of kin gave up the signed consent. Patients with septic shock diagnosed during ICU stay were eligible for the study. We used the American College of Chest Physicians/Society of Critical Care Medicine Consensus Conference definition of sepsis and septic shock [11]. Patients under 18 were excluded.

Severity of illness at the baseline was assessed based on the *acute physiology and chronic health evaluation II* (APACHE II) score [12]. Patients were assessed daily for 7 consecutive days using the *sequential organ failure assessment* score (SOFA) or until their discharge from the ICU when occurring in less than 7 days [13–15]. C-reactive protein and serum amyloid A protein were also measured daily.

The patients received conventional therapy regarding antibiotic regimens, serial blood cultures (whenever that body temperature >38°C), and discharge criteria. Relevant clinical and laboratory tests were conducted daily throughout the study.

Blood samples for CRP and SAA dosage were thawed and assayed in batches in an automated analyzer (Behring Nephelometer Analyzer II, Dade Behring, Marburg, Denmark) for particle-enhanced immunonephelometry using commercial kits. The analytical sensitivity and accuracy for CRP was 0.0175 mg/L (coefficient of variation (CV) 7,6%). The analytical sensitivity and accuracy for SAA was determined by the lower limit of the reference curve and thus depended on the concentration of the protein in SAA standard test (CV between 5.4% and 6.4%).

Statistical analysis was performed using commercial available package. Multiple logistic regressions were performed to test mortality of 7 or 28 days follow-up. A distribution analysis was made by the Kolmogorov-Smirnov test, Pearson correlation coefficients were determined, and repeated measures were tested by ANOVA. A *P* value < .05 was considered significant.

## 3. RESULTS

The mean (±SD) age of the 29 patients was 65 ± 13.9 years (range, 34 to 88 years). The study involved 13 males and 16 females (45%/55%). The APACHE II score of these patients was 19.8 ± 4.5 (Table 1). Table 2 represents the microbiological characteristics of the studied patients.

SOFA did not show increase from Day 0 to Day 7 of observation (*P* = .589, ANOVA) while CRP reduced significantly from Day 0 till the end of observation period (*P* < .001, ANOVA followed by Holm-Sidak test) as well as SAA (*P* < .001, ANOVA followed by Holm-Sidak test) (Table 3).

CRP and SAA concentrations did not present any correlation with SOFA.

On the other hand, CRP and SAA have shown a good correlation from Day 0 till Day 5 (Table 4).

Mortality of these patients in 7 days was 44.8% (13 in 29) and in 28 days was 65.5% (19 in 29). CRP and SAA concentrations were not associated with Day 7 mortality (Table 5).

**Figure 1:**
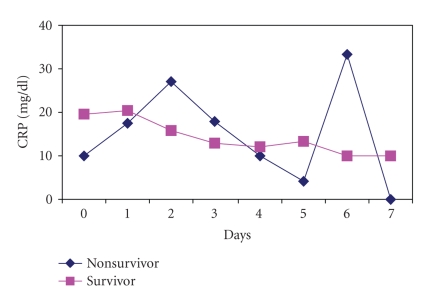
CRP evolution of survivors and nonsurvivors during the first week (NS). NS: not statistically significant.

**Figure 2:**
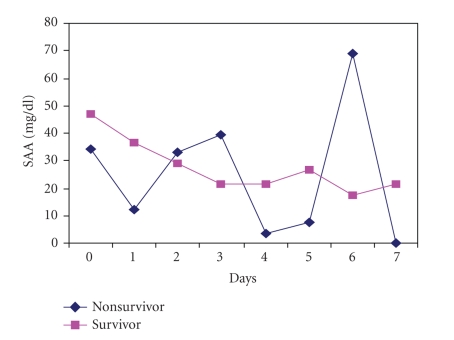
SAA evolution of survivors and nonsurvivors during the first week (NS). NS: not statistically significant.

CRP and SAA concentrations evolution during the first week comparing survivors and nonsurvivors were not statistically significant (Figures 1 and 2).

## 4. DISCUSSION

The present study revealed significant positive correlation between SAA and CRP in postoperative septic shock patients. SOFA or APACHE II did not correlate with those serum measurements. Neither marker nor index was associated with mortality rate.

SAA has been considered by some authors to be equivalent to CRP in patients with bacterial infectious diseases in clinical practice [16]. Other authors suggest that SAA is a more sensitive marker than CRP in infections with low inflammatory activity (including many viral infections) and in other clinical conditions, especially those involving the lung tissue [16, 17]. Yet other studies have confirmed the role of SAA and CRP in diagnosis and management of neonatal infections [2, 18].

The patterns of cytokine production and the acute-phase response differ for different inflammatory conditions. Acute-phase changes reflect the presence and intensity of inflammation and they have long been used as a clinical guide for diagnosis and management. Among patients with plasma CRP concentrations higher than 10 mg/dL, 80-to-85 percent have bacterial infections [4, 19]. In our study, all the patients were in septic shock with documented infection. We could observe that all patients during the 7-day period of observation presented plasma CRP concentrations greater than 10 mg/dL, according to results that Gabay et al. in a review article cited [4]. This fact could indicate that sepsis is secondary to bacterial infections. In relation to plasma SAA concentrations, a cutoff value has not been determined from previous studies. We observed a level higher or closer to 20 mg/dL, but more studies were needed to find a cutoff value for SAA as an early diagnostic tool for patients with infection.

We did not observe difference between CRP and SAA early (Day 1) concentrations in patients who survived compared with those who died. Other authors have found that these proteins were not prognostic markers in patients with septic shock [7, 19]. This fact is in concordance with our results.

Previous reports have observed that CRP level was associated with organ failure in critically ill patients, although not specifically under septic shock [14]. This study could not find any good correlation between CRP or SAA with SOFA, probably not in agreement with other authors [20]. They believe that both CRP and SAA are good markers of organ dysfunction, considering the established diagnostic of septic shock.

Study limitations are attributed primarily to the small sample size and the age of the patients that could influence CRP levels. Some authors believe that the older the patient is, the higher CRP levels that can be observed [21].

In conclusion, SAA protein and CRP are strongly correlated, but were not good predictors of organ dysfunction and mortality in septic shock.

## Figures and Tables

**Table 1:** Baseline characteristics of the patients.

**Characteristics**	**n = 29**
Age (years)	65 ± 13.9
Male sex	45%
Weight (kg)	63.5 ± 11.7
APACHE II score	19.8 ± 4.5
SOFA score	9.6 ± 2.3

**Prior or preexisting conditions**	(%)
Hypertension	31
Myocardial infarction	13.7
Diabetes	13.7
Liver disease	6.9
COPD	6.9
Cancer	20.7

**Surgery**	(%)
Multiple trauma (excluding head trauma)	3.4
Gastrointestinal surgery	75.9
Major vascular surgery	6.9
Thoracic surgery	3.4
Urologic surgery	10.4

**Other indicators of disease severity**	**(days)**
Mechanical ventilation	4.0 ± 3.2
Shock (use of vasopressor)	4.2 ± 1.9

*APACHE*: acute physiology and chronic health evaluation, *SOFA*: sequential organ failure assessment, COPD: chronic obstructive pulmonary disease.

**Table 2:** Microbiological characteristics of patients.

Patient	Surgery/phatology	Antibiotics	Type of organism	Type of culture
1	Cholecistectomy/biliary abscess	Vanco + cefepime	S. aureus	Abscess culture
2	Empyema pleural drainage	Ceftriaxone + clindamycin	S. pyogenes	Pleural abscess culture
3	Cholecistectomy/biliary abscess	Ceftriaxone + metronidazole	—	Negative cultures
4	Cystectomy/pyuria	Ceftriaxone + metronidazole	—	Negative cultures
5	Aortic bypass/leg amputation	Ceftazidime + clindamycin	P. aeruginosa	Surgical site culture
6	Colectomy/cavity contamination	Ceftriaxone + metronidazole	—	Negative cultures
7	Calcaneal exposure fracture	Ciprofloxacin	E. faecalis	Surgical site culture
8	Pyonephrosis drainage	Ceftriaxone	K. pneumoniae	Urinary culture
9	Sigmoidectomy	Ceftriaxone + metronidazole	A. baumanii	Blood culture
10	Hemicolectomy	Ceftriaxone + metronidazole	Candida albicans	Blood culture
11	Enterectomy/mesenteric ischemia	Ceftriaxone + metronidazole	—	Negative cultures
12	Pancreatic-duodenal resection	Ceftriaxone	Serratia marcesens	BAL
13	Pancreatic-duodenal resection	Ceftriaxone + metronidazole	S. coag negative	Blood culture
14	Retroperitoneal abscess drainage	Cefepime + vanco + imipenem	P. aeruginosa	Blood culture
15	Abdominal aneurysm repair	Vanco + imipenem	S. aureus	Blood culture
16	Sigmoidectomy/perforative lesion	Ceftriaxone + metronidazole	Serratia marcesens	Ascite culture
17	Colectomy	Cefepime + vanco	S. aureus	Blood culture
18	Gastric ulcer	Ceftriaxone + metronidazole	—	Negative cultures
19	Cholecistectomy	Cipro + metronidazole	Escherichia coli	Urinary culture
20	Hemicolectomy	Cefepime + vanco + metro	E. cloacae	Blood culture
21	Enterectomy/cavity contamination	Vanco + imipenem	—	Negative cultures
22	Colectomy	Ceftriaxone + metronidazole	A. baumanii	Blood culture
23	Colectomy	Ceftriaxone + metronidazole	P. aeruginosa	Blood culture
24	Enterectomy/cavity contamination	Ceftriaxone + metronidazole	—	Negative cultures
25	Cervical abscess drainage	Imipenem + vanco + metro	K. pneumoniae	Blood culture
26	Sigmoidectomy/perforative lesion	Ceftriaxone + metronidazole	P. aeruginosa	Blood culture
27	Sigmoidectomy	Cefepime + metronidazole	S. aureus	Blood culture
28	Pyonephrosis drainage	Cefepime + metronidazole	—	Negative cultures
29	Colectomy	Ceftriaxone + metronidazole	P. aeruginosa	BAL

Vanco: vancomycin, Cipro: ciprofloxacin, Metro: metronidazole, S. aureus: Staphylococcus aureus, S. pyogenes: Streptococcus pyogenes, P. aeruginosa: Pseudomonas aeruginosa, E. faecalis: Enterobacter faecalis, K. pneumoniae: Klebsiella pneumoniae, A. baumanii: Acinetobacter baumanii, S. coag negative: Staphylococcus coagulase negative, E. cloacae: Enterobacter cloacae, BAL: bronchoalveolar lavage.

**Table 3:** SOFA, CRP, and SAA during the study period (mean ± SD).

	Day 0	Day 1	Day 2	Day 3	Day 4	Day 5	Day 6	Day 7
SOFA	9.6 ± 2.3	10 ± 2.6	9.4 ± 4.1	9.8 ± 4.4	9.4 ± 4.2	9.9 ± 3.7	8.7 ± 4.1	8.6 ± 3.5
CRP	19.8 ± 8.4	20.9 ± 9.1	16.3 ± 6.2	13.2 ± 5.9	11.8 ± 7.7	12.7 ± 11.2	11.9 ± 8.3	10.0 ± 4.4
SAA	47 ± 39.8	37.2 ± 28.1	29 ± 21.48	22.7 ± 18.4	18.6 ± 20.8	24.7 ± 25.8	22.2 ± 20.5	21.7 ± 16.8

SOFA: sequential organ failure assessment, CRP: C-reactive protein, SAA: serum amyloid A. ANOVA. Equal variance test: SOFA *P* = .956, CRP *P* = .062, SAA *P* = .055.

**Table 4:** Pearson coefficient for SAA and CPR.

	*r*	*P*-value
Day 0	0.682	.0001
Day 1	0.660	.0004
Day 2	0.464	.034
Day 3	0.529	.024
Day 4	0.651	.0062
Day 5	0.778	.0028
Day 6	0.578	.628
Day 7	0.081	.822

**Table 5:** Maximum likelihood, Wald statistic (*P*-value).

	APACHE II	SOFA	CPR	SAA
Day 0	3.46 (.06)	2.46 (.11)	0.02 (.89)	1.91 (.17)
Day 1	3.06 (.08)	3.50 (.06)	1.01 (.31)	0.03 (.86)
Day 3	0.00 (.98)	0.00 (.98)	0.00 (.98)	0.00 (.98)

## References

[1] Luzzani A, Polati E, Dorizzi R, Rungatscher A, Pavan R, Merlini A (2003). Comparison of procalcitonin and C-reactive protein as markers of sepsis. *Critical Care Medicine*.

[2] Enguix A, Rey C, Concha A, Medina A, Coto D, Diéguez MA (2001). Comparison of procalcitonin with C-reactive protein and serum amyloid for the early diagnosis of bacterial sepsis in critically ill neonates and children. *Intensive Care Medicine*.

[3] Patel H, Fellowes R, Coade S, Woo P (1998). Human serum amyloid A has cytokine-like properties. *Scandinavian Journal of Immunology*.

[4] Gabay C, Kushner I (1999). Acute-phase proteins and other systemic responses to inflammation. *New England Journal of Medicine*.

[5] Urieli-Shoval S, Linke RP, Matzner Y (2000). Expression and function of serum amyloid A, a major acute-phase protein, in normal and disease states. *Current Opinion in Hematology*.

[6] Cicarelli LM, Perroni AG, Zugaib M, de Albuquerque PB, Campa A (2005). Maternal and cord blood levels of serum amyloid A, C-reactive protein, tumor necrosis factor-*α*, interleukin-1*β*, and interleukin-8 during and after delivery. *Mediators of Inflammation*.

[7] Ugarte H, Silva E, Mercan D, De Mendonça A, Vincent J-L (1999). Procalcitonin used as a marker of infection in the intensive care unit. *Critical Care Medicine*.

[8] Arnon S, Litmanovitz I, Regev R, Lis M, Shainkin-Kestenbaum R, Dolfin T (2002). Serum amyloid A protein in the early detection of late-onset bacterial sepsis in preterm infants. *Journal of Perinatal Medicine*.

[9] Nakayama T, Sonoda S, Urano T, Yamada T, Okada M (1993). Monitoring both serum amyloid protein A and C-reactive protein as inflammatory markers in infectious diseases. *Clinical Chemistry*.

[10] Reade MC, Young JD (2003). Consent for observational studies in critical care: time to open Pandora's Box. *Anaesthesia*.

[11] Levy MM, Fink MP, Marshall JC (2003). 2001 SCCM/ESICM/ACCP/ATS/SIS International Sepsis Definitions Conference. *Intensive Care Medicine*.

[12] Reny J-L, Vuagnat A, Ract C, Benoit M-O, Safar M, Fagon J-Y (2002). Diagnosis and follow-up of infections in intensive care patients: value of C-reactive protein compared with other clinical and biological variables. *Critical Care Medicine*.

[13] Hulley SB, Cummings SR (1988). *Designing Clinical Research*.

[14] Lobo SMA, Lobo FRM, Bota DP (2003). C-reactive protein levels correlate with mortality and organ failure in critically ill patients. *Chest*.

[15] Marshall JC, Vincent J-L, Fink MP (2003). Measures, markers, and mediators: toward a staging system for clinical sepsis. A Report of the Fifth Toronto Sepsis Roundtable, Toronto, Ontario, Canada, October 25-26, 2000. *Critical Care Medicine*.

[16] Lannergård A, Larsson A, Kragsbjerg P, Friman G (2003). Correlations between serum amyloid A protein and C-reactive protein in infectious diseases. *Scandinavian Journal of Clinical and Laboratory Investigation*.

[17] Huttunen T, Teppo A-M, Lupisan S, Ruutu P, Nohynek H (2003). Correlation between the severity of infectious diseases in children and the ratio of serum amyloid A protein and C-reactive protein. *Scandinavian Journal of Infectious Diseases*.

[18] Pizzini C, Mussap M, Plebani M, Fanos V (2000). C-reactive protein and serum amyloid A protein in neonatal infections. *Scandinavian Journal of Infectious Diseases*.

[19] Suprin E, Camus C, Gacouin A (2000). Procalcitonin: a valuable indicator of infection in a medical ICU?. *Intensive Care Medicine*.

[20] Castelli GP, Pognani C, Meisner M, Stuani A, Bellomi D, Sgarbi L (2004). Procalcitonin and C-reactive protein during systemic inflammatory response syndrome, sepsis and organ dysfunction. *Critical Care*.

[21] Wener MH, Daum PR, McQuillan GM (2000). The influence of age, sex, and race on the upper reference limit of serum C-reactive protein concentration. *Journal of Rheumatology*.

